# Triboelectric E-Skin Patch Decoding Silent Speech for Advanced Human–Machine Interaction and Augmented Virtual Communication

**DOI:** 10.1007/s40820-026-02346-x

**Published:** 2026-10-19

**Authors:** Ziwei Huo, Nuo Xu, Yixuan Fu, Rui Gu, Chao Liu, Zhong Lin Wang, Qijun Sun

**Affiliations:** 1 Beijing Key Laboratory of High-Entropy Energy Materials and Devices, Beijing Institute of Nanoenergy and Nanosystems, Chinese Academy of Sciences, Beijing, 101400 People’s Republic of China; 2 School of Nanoscience and Technology, University of Chinese Academy of Sciences, Beijing, 100049 People’s Republic of China; 3 Georgia Institute of Technology, Atlanta, GA 30332-0245 USA; 4 Center On Nanoenergy Research, School of Physical Science & Technology, Guangxi University, Nanning, 530004 People’s Republic of China

**Keywords:** Triboelectric sensor, E‑skin patch, Silent speech decoding, Human–machine interaction, Virtual communication

## Abstract

Silent speech decoding (lip reading) represents an innovative hands-free communication method for individuals with laryngeal and lingual impairments, addressing critical daily communication challenges. Herein, a novel silent speech decoding system is proposed, which employs a self-powered, low-cost, and conformable triboelectric-based E‑skin patch and a well-trained multi-layer convolutional neural network (CNN) model. We systematically characterize the structural principles and electrical performance of the E-skin patch. A comparative analysis of labial kinematics during vocalized and silent speech is also conducted, which demonstrates strong consistency in time–frequency domain characteristics. The utilized CNN model is trained across 15 lexical categories (200 samples per category), achieving a high accuracy of 98.75% and demonstrating excellent recognition performance. Furthermore, by integrating human–machine interaction (HMI) and virtual reality (VR) technologies, we develop three representative applications, including a real-time lip language visualization system, an orofacial-controlled “Feeding Frenzy” interactive game, and a VR communication platform with avatar dialogue. This technology enables barrier-free communication for voiceless populations, demonstrating significant potential in HMI, augmentative communication systems, virtual reality integration, and biomedical rehabilitation.

**Supplementary Information:**

The online version contains supplementary material available at https://doi.org/10.1007/s40820-026-02346-x.

## Highlights

- A triboelectric E-skin patch is developed to capture lip/facial muscle movements for silent speech decoding.
- The proposed four-layer convolutional neural network architecture assists to achieve a record high recognition accuracy of 98.75%.
- Featured human–machine interaction/virtual reality applications have been demonstrated, including silent speech display system, interactive game, and virtual communication.

**Supplementary Information:**

The online version contains supplementary material available at https://doi.org/10.1007/s40820-026-02346-x.

## Graphical abstract

**Figure:**
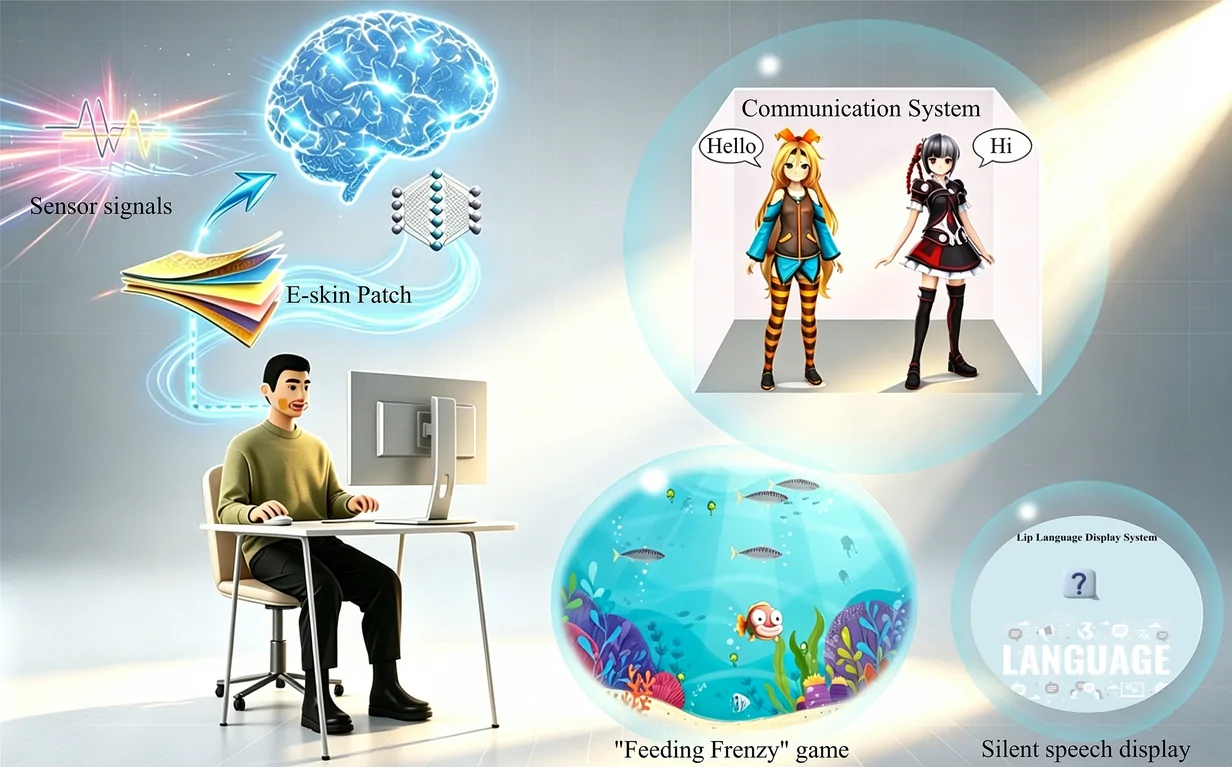

## Introduction

In human communication, facial expressions and lip movements serve not only as components of verbal communication but also as crucial expressions of emotion and intent. For individuals unable to use traditional vocal communication methods due to physiological or environmental constraints, technologies capable of capturing and interpreting these subtle signals are essential [1–3]. Advances in modern technology offer promising avenues to address this challenge. For instance, facial expression recognition technology can identify and interpret emotional states by analyzing facial muscle movements and eye interactions [4, 5], with applications spanning social robotics, intelligent monitoring systems, and medical diagnostics [6]. Leveraging machine learning (ML) [7] and computer vision algorithms [8, 9], such systems can learn to understand the meanings of various facial expressions, thereby enabling more intelligent human–machine interaction (HMI) [10, 11]. Nevertheless, the collection and analysis of personal facial expression data entail inherent risks, e.g., potential identity information leakage [12–14]. Additionally, cultural differences and the substantial volume of training data pose significant challenges for machine learning approaches in facial expression recognition tasks [15, 16]. Concurrently, lip-reading recognition technology has emerged as an important research field. By analyzing lip movement patterns, it recognizes and translates them into speech [17, 18], assisting individuals who are unable to vocalize or are in noisy environments to communicate effectively [19]. Lip-reading sensors, which analyze both lip movements and facial expressions, convert these non-verbal signals into comprehensible information [20], thereby bridging gaps in traditional voice communication and creating new communicative opportunities for users [21]. The application of lip-reading sensors is also expanding in daily life. For example, in virtual reality (VR) systems, capturing users’ lip movements allows for more realistic simulation of spoken communication, thereby enhancing the interactivity and immersion of the user experience [22–25]. Nevertheless, existing silent lip-reading techniques still confront prominent bottlenecks in practical implementation. Visual acquisition schemes suffer from privacy concerns and poor resistance to illumination changes and occlusion, while conventional wearable solutions are restricted by external power dependence and complicated system configuration [26–28]. In addition, distinct individual differences in lip movement patterns and pronunciation habits severely limit the cross-user generalization performance of recognition models [29–31]. Meanwhile, the growing number of recognition categories aggravates signal confusion, which raises higher requirements for effective feature extraction and accurate classification. In this context, it is urgent to explore self-powered flexible lip motion sensing solutions together with advanced feature learning strategies, which can get rid of external power supply, realize comfortable and privacy-friendly signal collection, and weaken individual motion differences to achieve reliable high-precision recognition of a massive vocabulary.

In recent years, triboelectric nanogenerators (TENGs) have attracted widespread global attention owing to their unique energy conversion mechanism based on contact electrification and electrostatic induction [32, 33]. TENGs can effectively harvest dispersed mechanical energy and directly convert it into electrical energy [34, 35]. This self-powered capability endows TENGs with considerable application potential across numerous fields [36, 37]. TENG-based sensors offer significant advantages of low-cost and self-powering, generating electrical signals without requiring an external power source, thereby greatly simplifying the design and deployment of sensing systems [38, 39]. Consequently, these sensors have demonstrated notable success in diverse applications. In healthcare, TENG-based sensors are employed for the real-time monitoring of human motion, physiological states, and environmental changes [40]. Tao Liu et al. recently reported a machine learning-assisted wearable sensing system for speech recognition and interaction, which captures laryngeal vibrations via a flexible acoustic sensor and achieves high-accuracy speech recognition even under noisy or masked conditions [41]. Shuai Lin et al. developed a self-powered triboelectric interactive interface dedicated to silent speech recognition, which also enables immersive interaction in augmented reality (AR) and VR scenarios [42]. Furthermore, TENG-based sensors exhibit broad application prospects in the field of HMI. Their exceptional sensitivity to various human motions—including finger tapping and even eye blinking—enables intuitive and natural interaction paradigms. Unlike traditional rigid interfaces, TENG-based HMI systems can be implemented on curved, stretchable, or even transparent substrates, opening new possibilities for smart surfaces, interactive displays, and wearable controllers [43–45]. This technological application facilitates more flexible and continuous device operation. Particularly in VR and AR, the deployment of TENG sensors enables more natural and tangible interactive experiences.

This paper proposes a flexible, low-cost, and self-powered electronic skin (E-skin) patch based on a TENG sensor. The device effectively captures orofacial muscle movements to decode silent speech with the assistance of deep learning, thereby enabling advanced HMI and augmented virtual communication. By capturing these muscle activities, the method offers a novel strategy for the direct and convenient conversion of lip movements into speech or text, providing potential assistance for individuals with speech impairments. Fabricated from flexible and low-cost polymer films, the self-powered E-skin patch is positioned at the most dynamic junction of the oral musculature. This placement enables the capture of subtle facial muscle movements and significantly enhances sensitivity during silent speech recognition. To address the inherent diversity and personalization challenges in silent speech, particularly within the context of limited training datasets, we introduce a multi-layer neural network model based on a convolutional neural network (CNN) architecture. This deep learning approach demonstrates excellent sensing performance, with the system achieving a record recognition accuracy of 98.75%, thereby validating its efficiency and accuracy. The developed triboelectric E-skin patch for silent speech decoding is readily adaptable to diverse living, working, and entertainment scenarios. Furthermore, we design an advanced silent speech display system that effectively captures triboelectric sensing signals from facial muscle movements (which may correspond to lip language in certain contexts) and innovatively converts them into intuitive visual graphics, clear text, and synthesized voice output. The utility of the self-powered E-skin patch is also demonstrated through an interactive electronic game (“Feeding Frenzy”), where decoded triboelectric signals serve as control commands. Moreover, an augmented virtual communication system is established by integrating the triboelectric E-skin patch with VR technology. This system allows an individual user to decode silent speech and control two virtual avatars to engage in real-time conversation. Compared with previously reported TENG-based silent speech systems that typically rely on multi-channel arrays or more complex recurrent neural networks (Table S1), our single-channel device achieves a state-of-the-art recognition accuracy of 98.75% using a lightweight multi-layer CNN architecture, significantly reducing system complexity and power consumption. This work presents a promising approach to facilitating barrier-free communication and enhancing the functional diversity of lip-reading translation systems within HMI and silent speech interfaces.

## Experimental Section

### Materials

Polyimide (Kapton) film (0.05 mm thickness) is purchased from DuPont Co., Ltd. (USA). Nylon film (25 μm thickness) and fluorinated ethylene propylene (FEP) film (0.08 mm thickness) were supplied by CG Co., Ltd. (China). Conductive silver fabric (0.1 mm thickness) is obtained from Longyang Electronics Co., Ltd. (China). Sponge (1 mm thickness) is purchased from Shenzhen Meixin Electronics Co., Ltd. (China). All materials were used as received without further purification.

### Preparation of the E-Skin Patch

The fabricated E-skin patch has overall dimensions of 6 × 6 cm^2^. Structurally, the device employed symmetric and uniformly sized FEP and nylon films, which served as the negative and positive triboelectric layers, respectively. To achieve stable and controllable contact-separation cycles between the friction layers, flexible sponge spacers with uniform width and thickness of 1 mm are evenly distributed along the device’s perimeter. This creates a consistent, stable gap structure between the positive and negative friction layers, ensuring full contact at the friction interface while enabling rapid separation under external excitation. Conductive silver fabric is tightly bonded to the outer surfaces of both friction layers as output electrodes, serving to collect and conduct the induced charge generated during triboelectric charging. Finally, the device is encapsulated with Kapton film to complete the fabrication of the E-skin patch.

### Analysis of the Relationship Between Silent Signals and Spoken Language

To quantitatively assess the correlation between silent lip-reading signals and vocalized speech, the phrase “hao jiu bu jian” is uttered at a normal speaking rate, during which the corresponding silent triboelectric voltage signals and audio signals are synchronously recorded. The envelope of the raw audio signal is first extracted using a sliding window maximum method. Using the onset of articulation as the temporal reference, the silent voltage signal is precisely aligned with the audio envelope to ensure complete temporal synchronization between the two signal sets. Subsequently, the linear correlation between the two signals is quantified using the Pearson correlation coefficient, which is calculated as follows:

1 $$r=\frac{{\sum}_{i=1}^{n}\left({x}_{i}-\overline{x}\right)\left({y}_{i}-\overline{y}\right)}{\sqrt{{\sum}_{i=1}^{n}{\left({x}_{i}-\overline{x}\right)}^{2}}\sqrt{{\sum}_{i=1}^{n}{\left({y}_{i-}\overline{y}\right)}^{2}}}$$

${x}_{i}$ and ${y}_{i}$ represent the sample values of the silent signal and the voiced signal at time step *i*, respectively; $\overline{x}$ and $\overline{y}$ are the mean values of the corresponding signals, and *n* is the number of valid sample points. Correlation is calculated based on *n* = 4000 sample points (sampling frequency 1 kHz, signal length 4 s), yielding a correlation coefficient of $r=0.94 \left(p<0.001\right)$. This indicates a strong linear correlation between the two sets of signals, demonstrating that the silent lip-reading signal can accurately characterize the temporal variation features of voiced speech.

### Characterization of the E-Skin Patch

Installed the E-skin patch on the two ends of a linear motor (Winnemotor) and a displacement stage with adjustable distance, the linear motor is controlled by LinMot software to accomplish cyclic contact-separation motions for the E-skin patch. The basic electrical outputs (open-circuit voltage, short-circuit current, and transferred charge) are measured by an electrometer (Keithley 6514) and recorded by a custom LabVIEW program.

### Data Collection and Annotation

Word signal acquisition was performed on three participants (two females and one male, aged 24–28 years) with the triboelectric E-skin patch attached to their faces. During the experiment, each participant silently articulated 15 target words (“blue”, “yellow”, “orange”, “purple”, “mutton”, “sausage”, “turkey”, “cucumber”, “bean”, “cabbage”, “grape”, “watermelon”, “breakfast”, “lunch”, and “dinner”) for ~ 70 times per word. To ensure sufficient data volume and statistical reliability, roughly 200 valid samples were collected for each word category, resulting in a total of 3000 samples. The voltage signals generated by the triboelectric E-skin patche were acquired using the electrometer at a sampling frequency of 1 kHz. Each valid sample is defined as the time window from the participant’s resting state (~ 0.5 s) prior to initiating the articulatory movement, through the completion of the articulation, and until the return to the resting state. All collected samples were manually annotated by word category, with each sample’s label corresponding one-to-one to the silently articulated word. The annotation process was independently reviewed by two experimenters to ensure accuracy. The complete dataset of 3000 samples was partitioned into a training set of 2400 samples and a test set of 600 samples, adhering to an 8:2 ratio. To mitigate the risk of overfitting due to data leakage, unique identifiers are assigned to each participant’s samples prior to the split. Stratified sampling is further employed to ensure that the proportion of data from each participant in both the training and test sets remained ~ 8:2, thereby effectively reducing evaluation bias that could arise from the repeated inclusion of individual participant characteristics.

### Integrated Systems and Relevant Applications

The E-skin patch is firmly fixed to the face area to accurately perceive and capture the voltage signals generated by the subtle lip movements during the articulation of words or sentences. During system development and testing, an Arduino UNO microcontroller (MCU) serves as the core control unit for stable signal acquisition and transmission. The virtual dialogue system is constructed and implemented on the Unity platform. Specifically, the platform reads the voltage signals detected and transmitted by the MCU through a dedicated serial port tool, ensuring real-time and reliable signal communication between the hardware device and the virtual system. System response time, a critical metric for real-time interaction performance, is evaluated by measuring end-to-end latency. The rising edge of the sensor output signal triggered by silent lip movements is defined as the starting point, whereas the completion of semantic recognition and execution of corresponding action feedback within the VR scene or HMI is defined as the endpoint. Both timestamps are recorded synchronously under a unified system clock, and the difference between them is calculated as the total latency. The results indicate that the time consumed at each stage is well-defined and stable. Specifically, the signal from the E-skin patch is acquired by the Arduino UNO via its analog-to-digital converter (ADC) at a sampling frequency of ~ 1 kHz, corresponding to an acquisition time of about 10 ms. The acquired data are then transmitted to a personal computer (PC) through a serial port, requiring ~ 5 ms. Subsequent processing—including data preprocessing, feature extraction, and response generation—is performed on the Python/Unity platform, with a total processing time of approximately 65 to 95 ms. This latency falls within an acceptable range for human–computer interaction, as users generally cannot perceive noticeable lag when the end-to-end latency remains below 100 ms. This performance satisfies the basic requirements of real-time interaction scenarios, including lip language display system, game control, and virtual communication.

## Results and Discussion

### Design and Characterizations of the Triboelectric E-Skin Patch

**Fig. 1:**
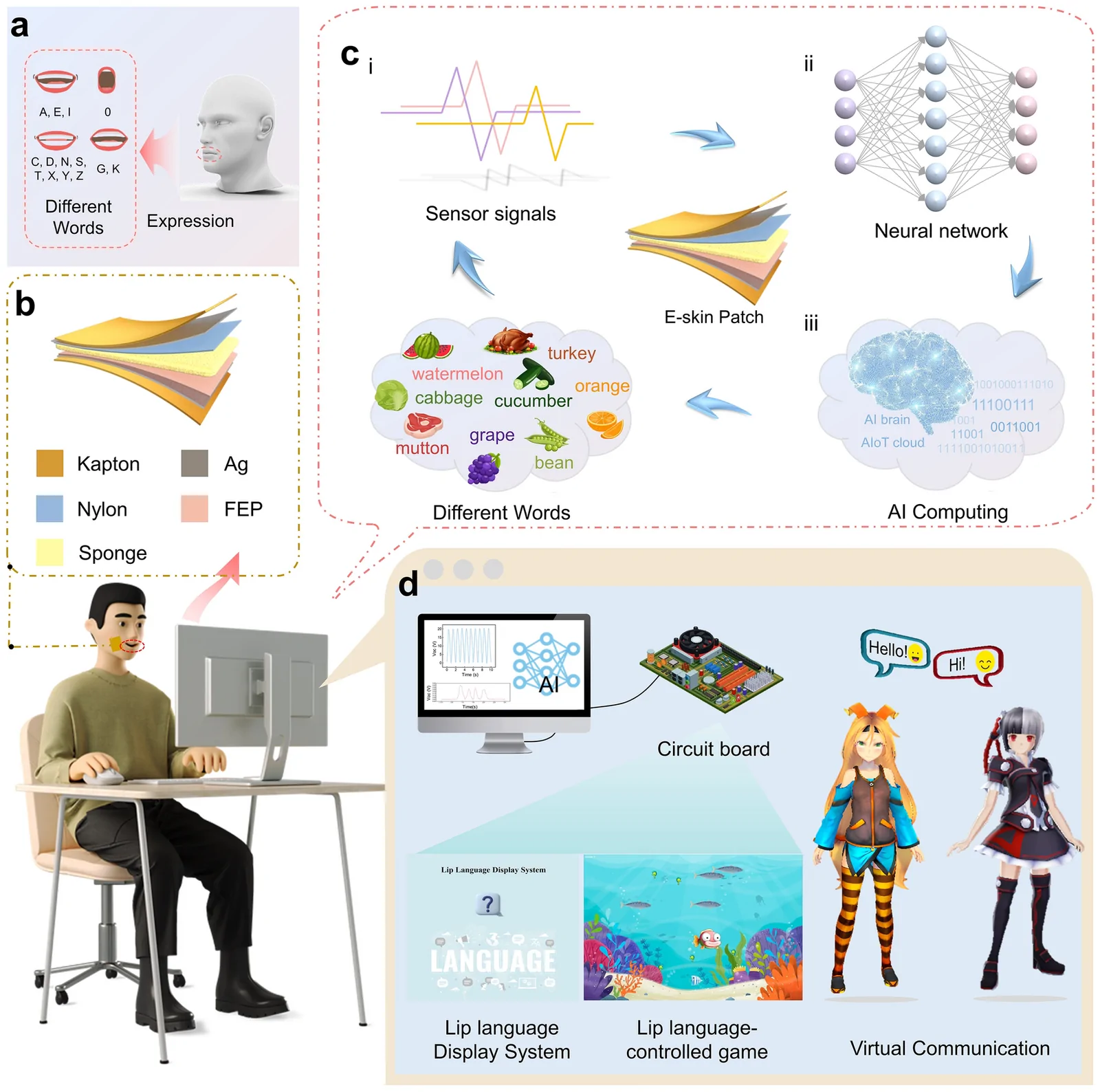
Concept, structure, and applications of the silent speech decoding systems based on the triboelectric E-skin patch. **a** Structure diagram of the triboelectric E-skin patch. **b** Schematic diagram of the application of the triboelectric E-skin patch in lip language. **c** Flowchart of the recognition and classification of 15 different words by a multi-layer neural network based on the CNN. **d** Applications of the E-skin patch include the lip language display system, a lip-controlled “Feeding Frenzy” game, and a virtual communication system.

As illustrated in Fig. 1a, the proposed silent speech decoding system based on a triboelectric E-skin patch effectively captures facial muscle movements to decode silent speech with the assistance of deep learning, thereby enabling advanced HMI and augmented virtual communication. During speech articulation, the wearable E-skin patch detects facial muscle activity and generates a sequence of triboelectric sensing signals. These electrical signals are captured, processed, and transmitted to a trained neural network for silent speech recognition and interaction identification. The flexible triboelectric E-skin patch (Fig. 1b), fabricated with fluorinated ethylene propylene (FEP) and Nylon film in contact-separation sensing mode, is positioned at the junction of orbicularis oris, depressor anguli oris, and buccinator to efficiently capture muscle movements. The primary materials used for triboelectric sensors are low-cost, readily available, and capable of conforming to facial muscle deformations, as illustrated by the photograph of the E-skin patch in Fig. S1. Upon signal acquisition, the triboelectric sensing data are initially visualized as pulse patterns. These pulse signals are subsequently processed by a neural network for feature extraction and pattern recognition, where intrinsic signal characteristics are decoded. Finally, the system converts the analytical results into interpretable semantic outputs (e.g., object-related vocabulary) in real time and high fidelity (Fig. 1c). This capability establishes the foundation for advanced HMI and augmented virtual communication applications (Fig. 1d).

**Fig. 2:**
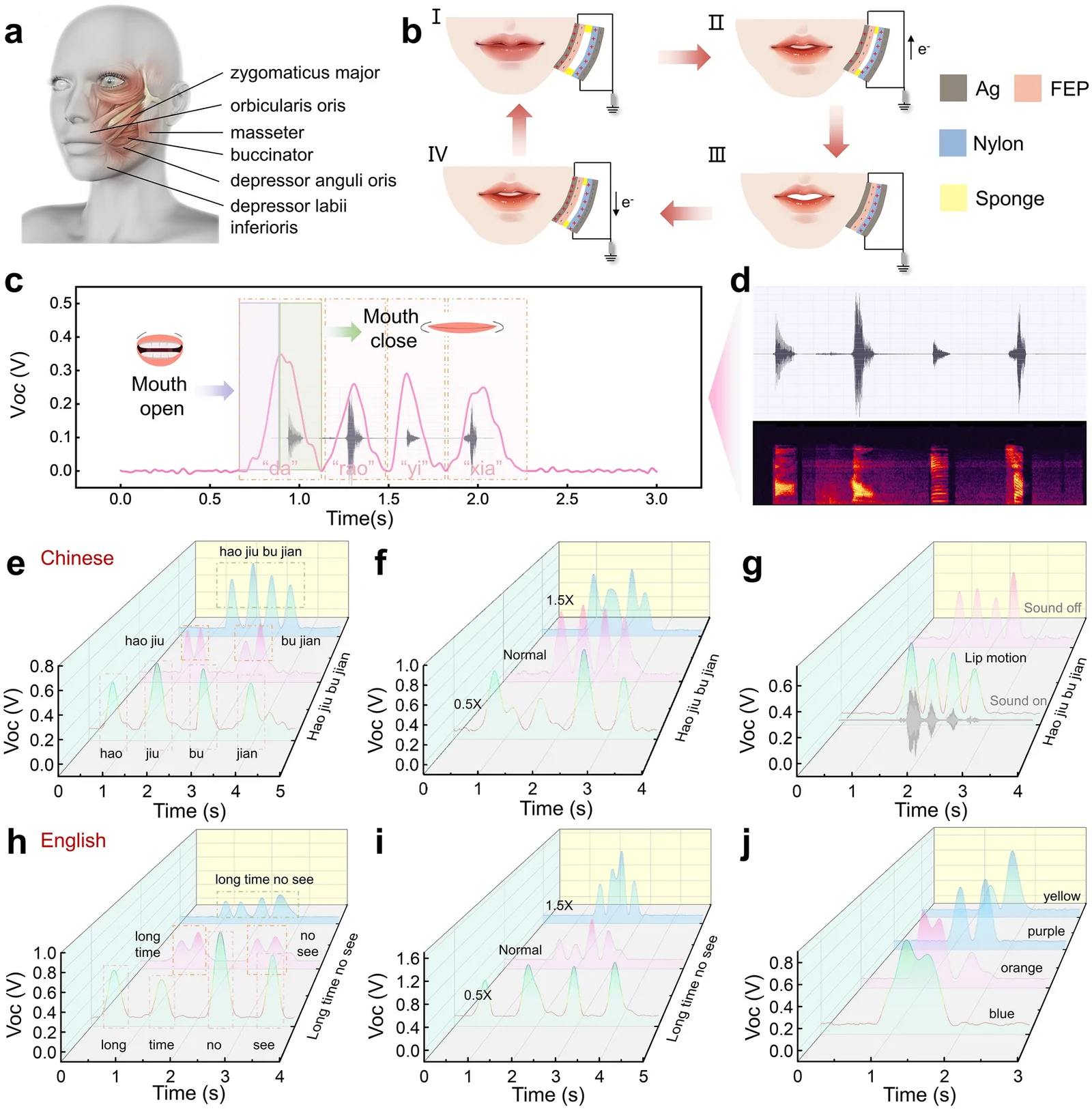
Basic electrical performance and sensing characterization of the E-skin patch. **a** Schematic diagram of the four stages of charge transfer in the mouth open-close cycle. The mouth-opening process pushes the sensor, and the mouth-closing process releases the sensor, resulting in the current flow in opposite directions. **b** Schematic diagram of the muscles associated with facial and lip control. **c** In a typical speech sequence (“da rao yi xia”), the mouth shape is synchronized with the signal. **d** Audio of the speaking sequence (“da rao yi xia”) and corresponding spectral features. **e** Combination and decomposition of lip movement signals of “hao”, “jiu”, “bu”, “jian”, “hao jiu”, “bu jian”, “hao jiu bu jian”. **f** Lip movement signals for “hao jiu bu jian” at different speech speeds (0.5 × normal, and 1.5 × speed). **g** Lip movement signals for silent and vocal speech, with simultaneously audio recording while saying “hao jiu bu jian”. **h** Combination and decomposition of lip movement signals of “long”, “time”, “no”, “see”, “long time”, “no see”, “long time no see”. **i** Lip movement signals for “long time no see” at different speech speeds (0.5 × normal and 1.5 × speed). **j** Distinct E-skin patch signal outputs are observed for various silent words, such as “blue”, “orange”, “purple”, and “yellow.” These four words show clear differences in voltage amplitude, waveform shape, and temporal response characteristics.

Facial (or labial) muscles collaborate with vocal cords to perform essential functions in speech production. Key muscles involved in facial movement during speech include the orbicularis oris, buccinator, and depressor anguli oris (Fig. 2a). The orbicularis oris encircles the lips, regulating their aperture for articulating distinct sounds. Supplementary muscles such as the zygomaticus major, masseter, and depressor labii inferioris further contribute to speech articulation. Coordinated activation of these muscle groups enables precise lip movements necessary for expressing thoughts and emotions. The triboelectric E-skin patch is strategically attached at the confluence of orbicularis oris, depressor oris, and buccinator muscles to capture muscle activity. Figure 2b details the dynamic sensing process, which comprises four distinct states based on charge transfer mechanisms. At the initial State I of speech resting, the FEP and Nylon films—serving as negative and positive friction layers, respectively—remain separated with no charge transfer. During facial muscle activation (State II), the FEP and nylon layers progressively approach, initiating electrostatic induction. Electrons flow from nylon to FEP through the external circuit. At peak muscular contraction (State III), the FEP and Nylon films achieve maximal contact (or proximity), generating peak sensing signals through optimal charge transfer. Subsequently, post-articulation muscle relaxation (State IV) triggers reverse electron flow through the external circuit until electrostatic equilibrium is restored.

To optimize the triboelectric E-skin patch for silent speech recognition, key device parameters—including friction layer materials, sensor dimensions, operating frequencies, and electrode materials—are systematically investigated using a linear motor and electrometer (Fig. S2). Their effects on device performance are presented in Figs. S3–S6. The output performance of the sensor is evaluated using three key electrical metrics: open-circuit voltage (*V*_OC_), short-circuit current (*I*_SC_), and transferred charge (*Q*_SC_). *V*_OC_ directly determines the signal amplitude generated by subtle lip movements. Higher *V*_OC_ improves signal strength and noise immunity. *I*_SC_ reflects charge migration rate and collection efficiency. Higher* I*_SC_ enables faster responses to lip movements, preserving waveform integrity and avoiding distortion. *Q*_SC_ governs total signal intensity and output stability per lip movement. Together, these electrical parameters determine the amplitude, waveform integrity, and stability of the sensor signal, which are critical for subsequent feature extraction and recognition. Smaller devices suffer low amplitude and severe waveform distortion due to insufficient coverage, making it impossible for lip-speech recognition (Fig. S7). The 6 × 6 cm^2^ device captures clear four-syllable waveforms, providing a reliable basis for feature extraction. Based on these systematic comparisons, we select the FEP friction layer, 6 × 6 cm^2^ device size, and 1 mm thickness of the spacer to construct the E-skin patch, which delivers the optimal electrical performance for capturing high-quality silent speech signals. To comprehensively characterize the device’s flexibility, additional bending performance tests are conducted, and photographs of the sensor under various bending conditions are provided (Fig. S8). The sensor is attached to cylindrical surfaces with different radii of curvature to evaluate the variation in output voltage with curvature radius (Fig. S9). The output voltage increases with decreasing radius of curvature due to closer triboelectric layer contact, confirming the e-skin’s excellent bending adaptability for conformal facial attachment.

Figure 2c demonstrates the optimized patch capturing signals during silent articulation of "da-rao-yi-xia" through lip motions. Notably, spectral analysis of vocalized audio signals for the same phrase (Fig. 2d) reveals a strong correlation with triboelectric signals during silent speech. Further analysis examines specific signal characteristics under varying silent speech conditions, including ‌ phonetic articulation‌, temporal dynamics, ‌and modality consistency. As shown in Fig. 2e, when one speaks the sentence of “hao-jiu-bu-jian” with phonetic articulation‌ (including “hao”, “jiu”, “bu”, “jian”, “hao jiu”, “bu jian”, and “hao jiu bu jian”), the results show that the output sensing signal of the triboelectric E-skin patch consistently matches facial movement cadence. In addition, we have compared the speaking rhythm and speed against audio baselines. Distinct signal patterns of “hao jiu bu jian” emerge at 0.5 ×, normal, and 1.5 × speed can be observed in Fig. 2f. As shown in Fig. S10a, the E-skin patch exhibits a response time of 90 ms and a recovery time of 65 ms, enabling rapid tracking of repetitive lip muscle movements to meet real-time signal acquisition requirements at normal speaking speeds. It achieves sensitivities of 3.70 kPa^−1^ (0–20 kPa) and 0.60 kPa^−1^ (20–60 kPa) (Fig. S10b), enabling sensitive detection of minute pressure changes and stable output across a wide pressure range. Furthermore, near-identical signatures between silent and vocalized “hao-jiu-bu-jian” reflect the modality consistency capability of the triboelectric E-skin patch (Fig. 2g).

Beyond the Chinese phrase “hao jiu bu jian”, we evaluated the English equivalent “long time no see” using the triboelectric E-skin patch under identical experimental conditions. During continuous orofacial movements corresponding to silent articulation of lexical components (“long”, “time”, “no”, “see”) and their combinations (“long time”, “no see”, “long time no see”) (Fig. 2h), the triboelectric signals exhibit precise temporal alignment with articulatory intervals. Figure 2i demonstrates velocity-dependent signal modulation during “long time no see” articulation at 0.5 ×, normal, and 1.5 × speech rates. The output waveforms consistently reflect movement frequency variations across speed conditions. Comparative analysis in Fig. S11 reveals strong correspondence between superimposed triboelectric and audio signals for both phrases at varying articulation rates. As shown in Fig. S12, the *V*_OC_ signals were recorded during the continuous articulation of the phrases “hao jiu bu jian” and “long time no see”, from which time–frequency heatmaps are computed after short-time Fourier transform (STFT). Simultaneously, the corresponding speech audio spectrograms are recorded. Comparative analysis reveals that the time–frequency energy distribution of the triboelectric signals exhibits high consistency with the audio spectrograms in the time–frequency domain. Furthermore, we examine word-specific articulation patterns (“blue”, “orange”, “purple”, “yellow”) as shown in Fig. 2j. Phonetic-driven fluctuations in labiomuscular activity directly correlate with signal amplitude and waveform characteristics, confirming the patch’s capacity to capture phoneme-specific articulatory kinematics. To further analyze the similarity and distinguishability of silent speech signals, the dynamic time warping (DTW) distance is employed as a quantitative metric. As shown in Fig. S13, the DTW distance between two repetitions of the Chinese phrase “hao jiu bu jian” is 1.42, substantially lower than that between “hao jiu bu jian” and the English phrase “long time no see” (2.71), indicating higher intra-class similarity. A DTW distance matrix for the 15 target words is further computed and visualized as a heatmap (Fig. S14). The diagonal (intra-class) distances are significantly smaller than the off-diagonal (inter-class) distances, demonstrating that the collected signals possess excellent repeatability, stability, and class separability, thereby providing effective feature support for deep learning models. Signal reproducibility is verified through ten repeated trials of the word “watermelon” (Fig. S15).

Repeated attachment and detachment tests over 30 cycles (Fig. S16a) show that the sensor retains 98% of its initial output after five cycles (gradually declines to 96% after ten cycles, 93% after twenty cycles, and 88% after thirty cycles), demonstrating good repeatability. Humidity tests under dry (~ 40% RH), mildly humid (~ 65% RH), and heavily humid (~ 85% RH) conditions (Fig. S16b) yield output voltages of 3.88 ± 0.08 V, 3.71 ± 0.09 V (95.6%), and 3.42 ± 0.11 V (88.1%), respectively. Although the signal amplitude decreases under high humidity, the output remains stable across all conditions and meets the requirements for reliable signal recognition. The 10,000-cycle test results (Fig. S17) indicate that the sensor output voltage exhibits no substantial decay, attesting to its exceptional long-term mechanical reliability.

### Deep Learning Assisted Silent Speech Recognition and Decoding

Lip language recognition is critical for enabling communication among individuals with dysphonia or articulation disorders. Our triboelectric E-skin patch facilitates facile and low-energy-consuming communication by rapidly analyzing orofacial movements. Given the complexity of daily communication and the intricate labiomuscular signals captured by sensors, deep learning algorithms are essential to ensure accurate and reliable silent speech recognition. CNNs require extensive labeled data for training, imposing substantial computational demands and overfitting risks. To address these limitations, we implement a multi-layer CNN architecture optimized for triboelectric signal recognition while minimizing computational overhead.

**Fig. 3:**
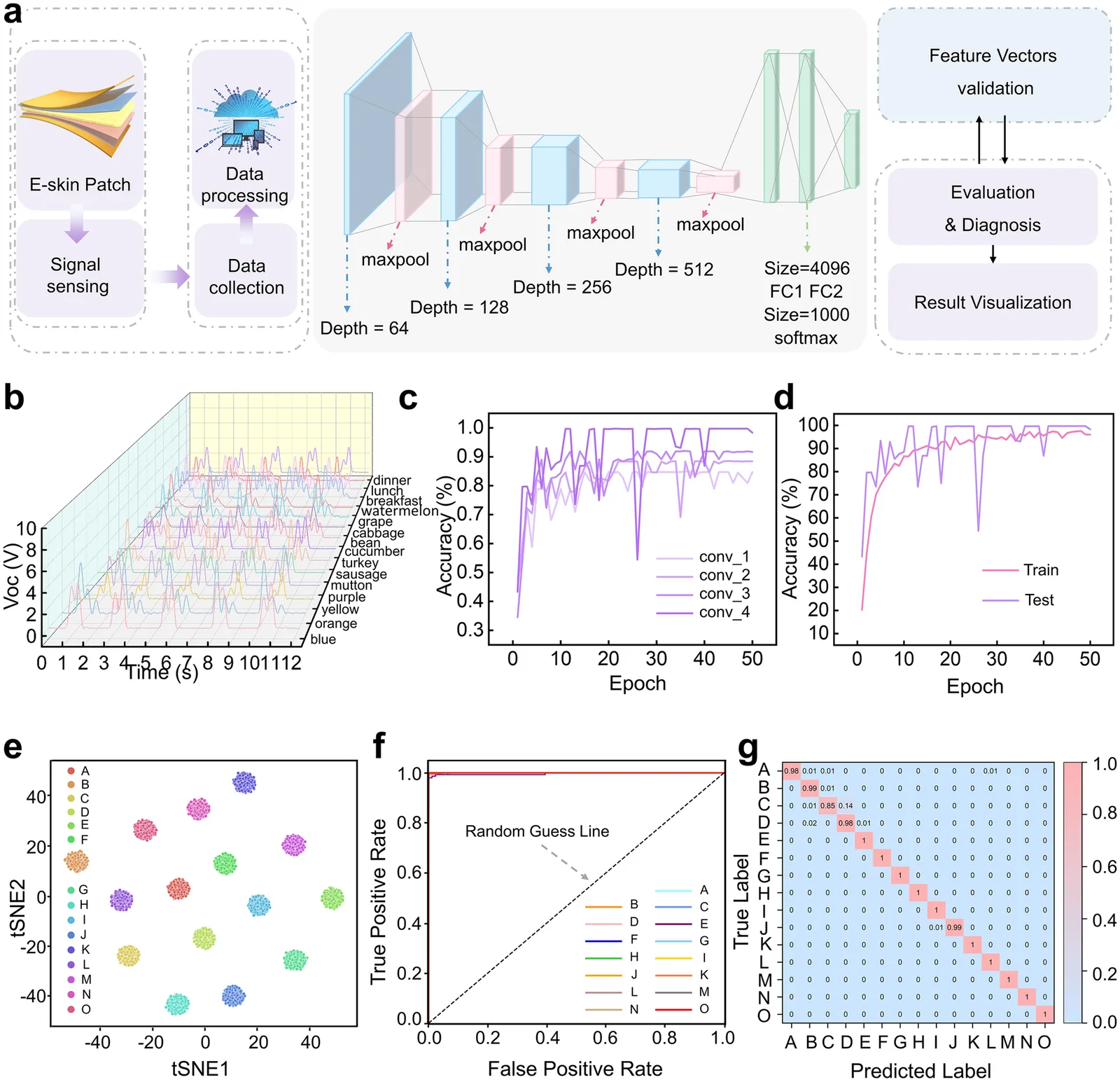
Schematic diagram of the multi-layer neural network structure based on CNN and classification results for 15 different words. **a** Multi-layer neural network structure diagram based on CNN and deep learning-assisted data processing flow. **b** Lip language signals for 15 different words. **c** Classification accuracy for different number of convolutional layers, with one to four convolutional layers corresponding to 84.9%, 88.6%, 91.89% and 98.75% accuracy, respectively. **d** Accuracy for 15 different words over 50 training epochs. **e** T-SNE clustering visualization of the 15 different words classifications via the multi-layer neural network. **f** ROC curve for the classification of the 15 different words by the multi-layer neural network. **g** Confusion matrix for classification of 15 different words by the multi-layer neural network.

Figure 3a illustrates the integrated sensing and training framework, in which the triboelectric E-skin patch positioned at the orbicularis oris-depressor anguli oris-buccinator junction captures labiomuscular motions during silent speech. The system acquires signals from 15 distinct words, with Fig. 3b demonstrating syllable-dependent waveform signatures and unique morphological characteristics. The sensing signals captured from the triboelectric E-skin patch are processed and encoded into feature vectors labeled with target semantic terms (e.g., Blue, Orange, Lunch, Dinner). Furthermore, STFT time–frequency analysis and audio spectral analysis are performed on each of the 15 target words (Figs. S18–S20). The syllables corresponding to the peak amplitudes in the time domain consistently exhibit distinct energy peaks in the frequency domain, with frequency ranges closely matching the acoustic characteristics of those syllables. Moreover, the dynamic temporal variations observed in speech are reflected as corresponding responses in the frequency domain, thereby providing a data foundation for subsequent word recognition based on acoustic features. To achieve better recognition performance, we adjust the CNN structure to process these vectors by modifying the number of filters and convolutional layers (four convolution layers with gradually increasing filter numbers (64, 128, 256, 512). Each convolutional layer is followed by a max-pooling layer for spatial downsampling and salient feature retention. After flattening, the features are fed into two fully connected layers (FC1: 4096 units; FC2: 1000 units), and a softmax layer outputs the probability distribution over semantic categories. The obtained feature vectors are validated for robustness, mapped to target words via regression, and finally evaluated to quantify the accuracy of semantic translation from the triboelectric sensing signals. The procedure for data acquisition and processing is illustrated in Fig. S21. We employ an early stopping strategy during training, halting the process when the loss on the test-set fails to decrease for five consecutive epochs. Additionally, we incorporate a dropout layer with a rate of 0.5 between the fully connected layers to enhance the model’s generalization ability. As evidenced in Fig. 3c, classification accuracy progressively increases with additional convolutional layers. This enhancement stems from the architecture’s capacity to extract hierarchical features—capturing finer details and higher-level semantic representations—which strengthens network learning capabilities while achieving higher recognition accuracy.

The proposed network, featuring four convolutional layers with filter counts incrementally increasing from 64 to 512 to extract hierarchical features, achieves a  recognition accuracy of 98.75% over 50 training epochs (Fig. 3d). The model is trained with a batch size of 32. For the four-layer CNN architecture, this relatively small batch size introduces substantial variance in gradient estimates during the later stages of training, particularly as the model approaches convergence. Such gradient noise manifests as high-frequency fluctuations in test set accuracy, which directly corresponds to the spike-like oscillations observed in the early training phase and the minor fluctuations evident in the later phase. A stepwise learning rate decay strategy is employed, reducing the learning rate from 0.001 to 0.0005 at epoch 20 and further to 0.00025 at epoch 30. Following each decay, the model’s sensitivity to gradient noise increases significantly, accompanied by a corresponding increase in the amplitude of test accuracy fluctuations. This trend closely matches the heightened oscillation amplitude observed between epochs 20 and 30. Furthermore, although the total dataset comprises 3000 samples, the average number of samples per category is only 200 across the 15 word classes. This relatively limited dataset size leads to unstable parameter updates when the model processes small batches during the later stages of training, thereby giving rise to minor fluctuations in test accuracy. To further validate the stability of the results, twelve additional experiments are conducted using different random seeds. The training and testing accuracy curves for each experiment are presented in Fig. S22, and the final results are summarized in Table S2. Across all twelve experiments, the test set accuracy exhibits a consistent pattern characterized by a rapid initial increase, followed by fluctuations during the mid-phase, and finally minor oscillations at a sustained high level in the late phase. This consistency indicates that the observed phenomenon is an inherent characteristic of the training process rather than a random outcome of a single experiment. After convergence at the 50th epoch, the test set accuracy across the twelve experiments ranges from 98.575% to 99.671%, with a mean accuracy of 98.892% and a standard deviation of only 0.335%. The difference between test accuracy and training accuracy is minimal, with no systematic downward trend, demonstrating that the model’s generalization performance remains stable and that the observed fluctuations do not adversely affect the final performance. To visualize clustering patterns of the word dataset, we apply t-distributed stochastic neighbor embedding (t-SNE), reducing high-dimensional representations to a 2D space. This scatter plot (Fig. 3e) displays each word  sample as a distinct point, with color-coded categories illustrating clear inter-class separation. Labels A–O correspond to the following categories: blue, yellow, orange, purple, mutton, sausage, turkey, cucumber, bean, cabbage, grape, watermelon, breakfast, lunch, and dinner. Specifically, 30 samples are randomly selected from the test set for each word category, resulting in a total of 450 points for dimensionality reduction and plotting. Visualizations based on the test set provide a more accurate assessment of the model’s ability to discriminate features on unseen data. The results demonstrate that features from different word categories remain well-clustered even when evaluated on the test set, indicating the absence of significant overfitting. For classification performance evaluation, receiver operating characteristic (ROC) curve analysis is conducted. Here, the false positive rate (FPR) measures the misclassification of negatives, while the true positive rate (TPR) quantifies the correct positive identification. A higher area under the curve (AUC) indicates superior model performance. As shown in Fig. 3f, the ROC curves demonstrates high efficacy of our multi-layer CNN in classifying the 15 words. Additionally, Fig. 3g visualizes classification outcomes via a confusion matrix, confirming the model’s robust discriminative capability across diverse semantic categories.

To further validate the model’s generalization ability, a subject-independent evaluation is conducted. Four participants (two males and two females) are recruited (Fig. S23a). The model is evaluated on each participant’s independent dataset, and a cross-subject test is performed by training on three participants and testing on the fourth. These results demonstrate that the model effectively overcomes individual differences and achieves high-precision recognition on unfamiliar users, exhibiting excellent cross-subject generalization capability. To evaluate long-term stability, cross-day tests are conducted (Fig. S23b). The accuracy shows only a slow, slight decline over time, consistently remaining above 97.6%. These findings demonstrate that the proposed triboelectric E-skin patch possesses excellent long-term stability and cross-temporal robustness. To validate the advantages of the proposed multi-layer CNN architecture, a systematic comparison is conducted with traditional machine learning models (SVM, Random Forest, KNN), recurrent neural networks (LSTM, GRU), and Transformer models on the same dataset (Table S3). The results demonstrate that the proposed CNN achieves superior accuracy, training efficiency, and model complexity, offering significant advantages for silent lip-reading decoding. To evaluate long-sentence decoding performance, five representative English sentences (three to nine words in length) are selected. Two hundred samples are collected per sentence, and end-to-end recognition is performed using the same CNN model as in the 15-word recognition task. As shown in Fig. S24, the system achieves an average recognition accuracy of 97.3%, validating its effectiveness and robustness for long-sentence decoding.

### Triboelectric E-Skin Patch Assisted Human–Machine Interaction

Advances in artificial intelligence (AI) and ML have significantly enhanced the intelligence and adaptability of HMI systems [46–48]. Integrating lip-reading recognition with HMI creates novel communication pathways for non-verbal users, substantially improving the quality of life for individuals with speech disabilities. By leveraging triboelectric sensors to decode labiomuscular movements into silent speech information, these systems represent a critical breakthrough in overcoming communication barriers. They demonstrate adaptive learning capabilities for personalized orofacial patterns, enabling precise command execution via subtle lip gestures.

**Fig. 4:**
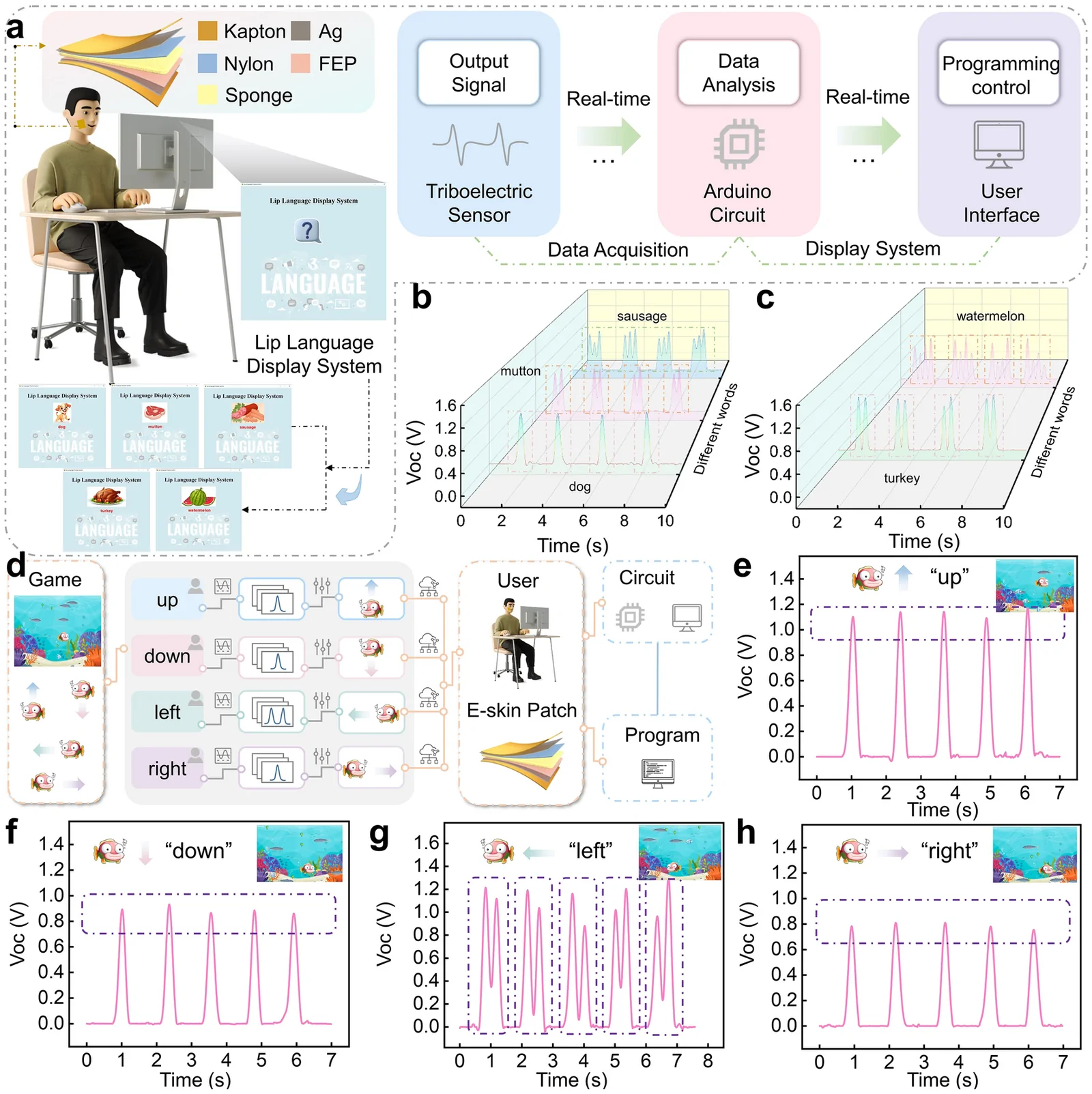
Triboelectric sensor-controlled lip language display system and the “Feeding Frenzy” game. **a** Schematic diagram of the data acquisition and processing workflow of the lip language display system. **b** Lip language signals corresponding to the words “dog”, “mutton” and “sausage”. **c** Lip language signals for the words “turkey” and “watermelon”. **d** Schematic diagram of the “Feeding Frenzy” game workflow controlled by “up”, “down”, “left”, and “right” lip language signals. **e** “Up”, **f** “down”, **g** “left”, and **h** “right” lip language signals control the swimming of the big fish according to the differences in the amplitude of the signals.

Figure 4a illustrates our triboelectric E-skin patch integrated silent speech display system. The patch detects subtle orofacial movements and converts them into electrical signals, while an Arduino microcontroller enables real-time data transmission to Python-based processing modules. This system receives and decodes the triboelectric sensing signal, where, for the silent speech display system, a template-matching rule-based algorithm is adopted. Specifically, standard signal templates for each word is pre-collected, and the real-time acquired signals are then matched with the templates through cross-correlation calculation to achieve recognition, thereby identifying semantic content‌ through preset algorithms, and ‌renders outputs‌ as visual/textual displays. This intelligent processing method enables intuitive and prompt display of the facial/mouth/lip motion information on the system interface, offering a convenient and efficient communication tool for users and recipients. The synergistic operation of the E-skin patch, Arduino board, and Python program achieves precise silent speech recognition, real-time information conversion, and intuitive interface visualization. Validation tests (Fig. 4b, c) demonstrate accurate signal capture and transformation for lexical items (“dog”, “watermelon”, etc.) into corresponding pictorial representations (Video S1), confirming the system’s efficacy in bridging non-verbal communication gaps.

To enhance immersive entertainment for individuals with speech disorders, we develop a triboelectric E-skin patch-controlled interactive game “Feeding Frenzy”. Players navigate a large fish using orofacial movements, with directional commands (up/down/left/right) mapped to specific muscle activations. Figure 4d illustrates the interactive game process: the Arduino microcontroller acquires real-time sensing signals, which are processed into directional commands via a Python program to control the fish’s movement within an underwater scene. As the large fish consumes a small fish, its position and score are dynamically updated. As shown in Fig. 4e–h, the triboelectric E-skin patch generates distinct voltage amplitudes corresponding to the four directional commands. Notably, the voltage signals of the four commands exhibit easily distinguishable characteristics: the three monosyllabic commands (“up”, “down”, “right”) each display a single-peak waveform with significantly different peak voltages (~ 1.1 V for “up”, ~ 0.9 V for “down”, and ~ 0.7 V for “right”), while the disyllabic command "left" generates a distinct multi-peak burst waveform, fundamentally differing in morphology from the single-peak ones. These differential signatures originate from movement complexity: single-syllable commands require isolated muscle contractions, while disyllabic "left" necessitates sequential buccinator-orbicularis oris coordination. For the ‌recognition algorithm, we leverage the captured unique signal characteristics to realize precise command implementation. Specifically, we design a lightweight rule-based algorithm: voltage amplitude thresholds were used to distinguish the three monosyllabic commands, and a peak-counting algorithm is employed to identify the multi-peak pattern of "left" command. This algorithm achieves great classification accuracy for the four-directional command set, with extremely low computational overhead and minimal response latency—key advantages making it particularly suitable for real-time interactive gaming scenarios. We set voltage amplitude thresholds to distinguish single-syllable of “up”, “down”, and “right”, and use a peak-counting algorithm to identify the multi-peak pattern of “left”. This approach ensures accurate classification of lip signals, immediate game response, and smooth execution of directional commands, delivering an immersive gaming experience. Precise and smooth game control of “Feeding Frenzy” via the triboelectric E-skin patch is further demonstrated in Video S2, highlighting the broad application potential of triboelectric sensors in HMI systems.

### Triboelectric E-Skin Patch Assisted Virtual Communication

VR technology delivers immersive experiences that transform human interactions with virtual spaces. Emerging VR interfaces have found extensive applications in diverse fileds, enhancing interactivity and immersion [49–51]. Crucially, users in VR environments can control character dialogues solely through orofacial movements during silent speech, enabling active participation in virtual experiences. This positions VR as an ideal platform for developing advanced lip-reading interfaces that facilitate information visualization and communication.

**Fig. 5:**
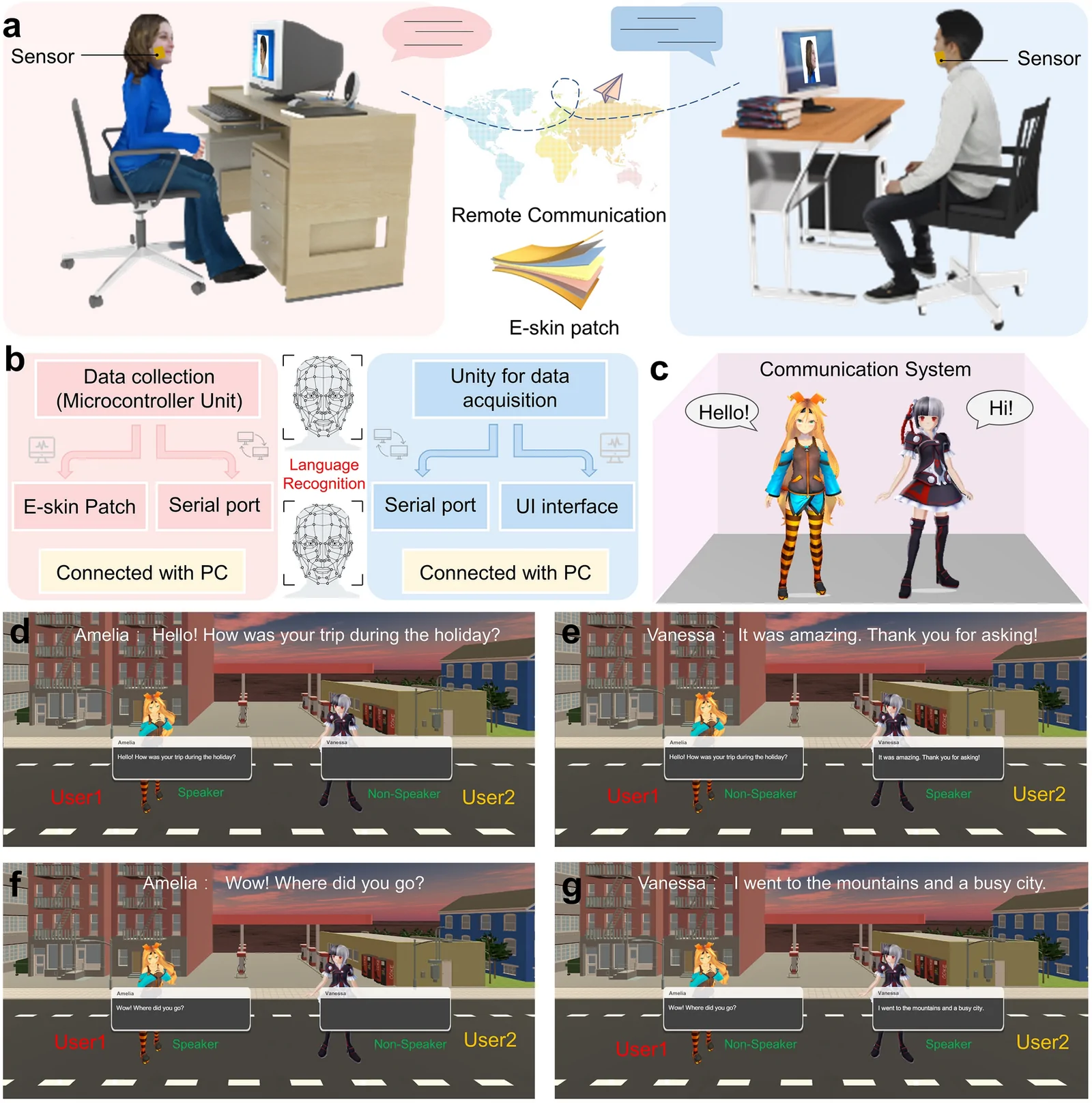
Virtual communication system controlled by the E-skin patch. **a** Remote communication application based on triboelectric sensors that detect lip language signals for control. Signals collected by the E‑skin patch are transmitted via the serial port of the microcontroller unit to Unity. The received voltage signals are then used to implement virtual dialogue on the user interface (UI). **b** Data acquisition and control flowcharts for the virtual communication system. **c** Schematic diagram of the virtual communication system. **d** Dialogue of character Amelia, controlled by the triboelectric sensor: “Hello! How was your trip during the holiday”. **e** Dialogue of character Vanessa: “It was amazing. Thank you for asking!” **f** Question from character Amelia: “Wow! Where did you go” and **g** answer from character Vanessa: “I went to the mountains and a busy city”.

Figure 5a illustrates our virtual communication system integrating the triboelectric E-skin patch for silent speech decoding, which enables silent communication to convey clear information. This platform enables clear information exchange through three core modules, including ‌orofacial motion capture with high-sensitivity triboelectric sensor at optimized anatomical positions, and data acquisition‌ via an Arduino-connected PC with analog signal processing (Fig. 5b). Specifically, the E-skin patch is attached to the optimized position to precisely capture the subtle orofacial kinematics. The triboelectric sensing signals can be converted into multimodal outputs (text/audio), transmitted to virtual environments via interactive protocols, and realize programmatic translation through Unity analysis modules. When a user wearing the E-skin patch performs silent lip movements, the sensor converts the associated muscle activity into electrical signals. These signals are acquired by an Arduino microcontroller and transmitted through a serial port. The Unity platform then recognizes the input signals using predefined algorithms, decoding them into corresponding text. This text is subsequently fed into a text-to-speech (TTS) module integrated into Unity, which invokes the system’s built-in speech synthesis engine (the Unity TTS API) to convert the text into synthesized speech with a preset voice and play it in real time. Simultaneously, the text is displayed as a speech bubble for the virtual character. When another user responds, the sensor worn by that user repeats the same process of signal acquisition, recognition, and TTS generation, thereby completing a bidirectional virtual dialogue interaction.

The face/mouth/lip movement recognition is demonstrated in an actual scene with two test characters wearing the triboelectric E-skin patch. The relevant VR interfaces are designed for communication between the two users with language barriers, switching between host and guest perspectives in cyberspace. As shown in Fig. 5c, the two virtual characters (“Amelia” and “Vanessa”) are established with controlled and programmable multi-degree of freedom movement in a virtual reality space, representing the two users with speech impediments. To further validate the system’s practicality and reliability, real-time VR dialogues are tested. Real-time sensor signals are collected for each sentence, and DTW analysis is performed on 29 individual words and four complete sentences, generating DTW heatmaps (Figs. S25–S27). The results demonstrate that sensor signals exhibit characteristic peaks highly synchronized with syllable structure, with waveform features differing significantly across sentences. All signals remain continuous and stable, with no degradation or drift, confirming the sensor’s reliability in continuous conversational scenarios. Furthermore, intra-class DTW distances for words (0.5–2.1) are significantly lower than inter-class distances (2.2–9.0), and intra-class distances for sentences are below 1.0 versus inter-class distances above 1.9, indicating high feature distinguishability without cross-interference.

As shown in Fig. 5d, when Amelia silently queries "Hello! How was your trip during the holiday?”, the orofacial motions are successfully captured by the triboelectric E-skin patch, which are then converted into sensing signals and recognized by the Unity program to implement a virtual dialogue system with readable text and audible audio. In Fig. 5e, another user Vanessa responds: “It was amazing. Thank you for asking!” The communication system also subtly transforms this response into text and sends it back to Amelia in the form of speech, thus achieving a smooth and emotionally rich communication experience between the two users. Subsequently, the two users continue the conversation with Amelia asking "Wow! Where did you go” (Fig. 5f) and Vanessa replying “I went to the mountains and a busy city” (Fig. 5g). The dialogue and real-time scenario in which two characters engage in the virtual communication are shown in Video S3. The virtual communication system, which combines the triboelectric E-skin patch, VR, and communication technology, demonstrates pioneering significance for future barrier-free remote communication, assistive education for hearing-impaired communities, and next-generation accessible HMIs.

## Conclusions

In conclusion, this study demonstrates a silent speech recognition and interaction system utilizing a triboelectric E-skin patch. The wearable patch, affixed to the user’s face, effectively captures orofacial movements and transmits corresponding electrical signals to a decoding system for conversion into intelligible communication outputs. We characterize the working mechanisim along with the mechanical and electrical properties of the E-skin patch, and analyze signal acquisition dependencies on speech velocity and labial kinematics. A multi-layer CNN architecture achieves 98.75% recognition accuracy across 15 semantic categories using 3,000 test samples. The system’s applicability is extended to diverse scenarios, including an ‌instantaneous lip-reading display system‌ converting signals into multimodal outputs (text/audio/visuals), an ‌interactive game‌ (“Feeding Frenzy”) controlled by orofacial commands, and a ‌VR communication platform‌ enabling natural dialogue via silent speech. This work advances assistive technologies for speech-impaired individuals, demonstrating significant potential in HMI, augmentative and alternative communication, rehabilitation engineering, biomedical interfaces, and immersive virtual environments.

Future work will focus on integrating the self-powered E-skin patch with eye trackers and brain-computer interfaces for multimodal assistive systems. Advances in low-power edge AI chips (e.g., TinyML) will enable on-device CNN inference, thereby improving portability, real-time performance, and data privacy. Additionally, device miniaturization will be pursued through optimized electrode patterning (e.g., serpentine or grid structures) and more efficient charge collection strategies. Coupling the system with large language models could enable intelligent dialogue that decodes lip movements, understands intent, and generates responses. Sensor sensitivity and output performance can be further enhanced by exploring high-dielectric-constant and high-charge-density materials, such as surface-functionalized polymers and composite dielectrics.

## Supplementary Information

Below is the link to the electronic supplementary material.

Supplementary file1 (MP4 1777 kb)

Supplementary file2 (MP4 4857 kb)

Supplementary file3 (MP4 2314 kb)

Supplementary file4 (DOCX 27615 kb)
