# Supplementary material for: Triboelectric E-Skin Patch Decoding Silent Speech for Advanced Human–Machine Interaction and Augmented Virtual Communication
Source: Nanomicro Lett. 2026 Oct 19;19:55. doi: 10.1007/s40820-026-02346-x (PMC13677660; doi:10.1007/s40820-026-02346-x)
Supplement: Supplementary file 1 [file 40820_2026_2346_MOESM4_ESM.docx]

Supporting Information for

**Triboelectric E-Skin Patch Decoding Silent Speech for Advanced Human-Machine Interaction and Augmented Virtual Communication**

Ziwei Huo^1, 2^, Nuo Xu^1, 2^, Yixuan Fu^1, 2^, Rui Gu^1, 2^, Chao Liu^1, 2^, Zhong Lin Wang^1, 3, *^, Qijun Sun^1, 2, 4, *^

^1^ Beijing Key Laboratory of High-Entropy Energy Materials and Devices, Beijing Institute of Nanoenergy and Nanosystems, Chinese Academy of Sciences, Beijing 101400, P. R. China

^2^ School of Nanoscience and Technology, University of Chinese Academy of Sciences, Beijing 100049, P. R. China

^3^ Georgia Institute of Technology, Atlanta, Georgia 30332-0245, United States

^4^ Center on Nanoenergy Research, School of Physical Science & Technology, Guangxi University, Nanning 530004, P. R. China

*Corresponding authors. E-mail: [sunqijun@binn.cas.cn](mailto:sunqijun@binn.cas.cn) (Qijun Sun); [zhong.wang@mse.gatech.edu](mailto:zhong.wang@mse.gatech.edu) (Zhong Lin Wang)

**S1 Supporting Figures and Tables**

**Fig. S1** Digital photographs of the flexible E-skin patch. Front view (left) and side view (right) demonstrating the flat, thin profile and excellent flexibility of the device.

**Fig. S2** Schematic diagram of the testing process for electrical properties of the E-skin patch. The linear motor is used to collect force signals for sensor performance characterization, while facial/lip movements corresponding to various phonemes during silent speech are captured by flexible triboelectric sensors. Both force and triboelectric signals are acquired by an electrometer and then transmitted to a computer for real-time visualization and subsequent analysis.

**Fig. S3** Open-circuit output voltage (*V*_OC_) of the E-skin patch under different parameters. **(a)** *V*_OC_ of the E-skin patch under different friction layer materials, showing a clear material-dependent triboelectric performance, where FEP yields the highest peak voltage (~20 V) due to its strong electron-withdrawing ability. **(b)** *V*_OC_ of the E-skin patch with different contact areas. The peak voltage monotonically increases with the contact area, rising from ~15 V (3×3 cm²) to ~40 V (6×6 cm²), indicating a positive correlation between effective contact area and triboelectric charge generation. **(c)** *V*_OC_ of the E-skin patch at different frequencies from 1 Hz to 3 Hz. **(d)** *V*_OC_ of the E-skin patch with different spacer thicknesses from 1 mm to 5 mm. **(e)** *V*_OC_ of the E-skin patch with different spacer widths from 1 mm to 4 mm. **(f)** *V*_OC_ of the TENG sensor under different pressures from 1 N to 4 N.

**Fig. S4** Short-circuit output current (*I*_SC_) of the E-skin patch under different parameters. **(a)** *I*_SC_ of the E-skin patch under different friction layer materials. **(b)** *I*_SC_ of the E-skin patch with different contact areas. **(c)** *I*_SC_ of the E-skin patch at different frequencies from 1 Hz to 3 Hz. The peak current increases linearly with frequency, from ~20 nA (1 Hz) to ~130 nA (3 Hz), while the signal repetition rate increases, reflecting enhanced charge transfer efficiency at higher motion speeds. **(d)** *I*_SC_ of the E-skin patch with different spacer thicknesses from 1 mm to 5 mm. The peak current gradually decreases with increasing spacer thickness, dropping from ~40 nA (1 mm) to ~15 nA (5 mm), as larger gaps reduce the efficiency of triboelectric charge separation and electrostatic induction. **(e)** *I*_SC_ of the E-skin patch with different spacer widths from 1mm to 4 mm. The peak current declines monotonically with increasing spacer width, from ~45 nA (1 mm) to ~20 nA (4 mm), demonstrating that wider spacers limit the effective contact area and charge accumulation. **(f)** *I*_SC_ of the E-skin patch under different pressures from 1 N to 4 N.

**Fig. S5** Short-circuit transferred charge (*Q*_SC_) of the E-skin patch under different parameters. **(a)** *Q*_SC_ of the E-skin patch under different friction layer materials. **(b)** *Q*_SC_ of the E-skin patch with different contact areas. **(c)** *Q*_SC_ of the E-skin patch at different frequencies from 1 Hz to 3 Hz. **(d)** *Q*_SC_ of the E-skin patch with different spacer thicknesses from 1 mm to 5 mm. **(e)** *Q*_SC_ of the E-skin patch with different spacer widths from 1 mm to 4 mm. **(f)** *Q*_SC_ of the E-skin patch under different pressures from 1 N to 4 N. The peak short-circuit transferred charge increases linearly with the magnitude of the applied force, rising from ~8 nC (1 N) to ~16 nC (4 N), which confirms that higher pressure enhances interfacial contact tightness and thus increases the total amount of transferred charge per cycle.

**Fig. S6** Comparation of the electrical output of the triboelectric devices with different electrode materials. **(a)** *V*_OC_, **(b)** *I*_SC_, and **(c)** *Q*_SC_ of the devices using Ag, Cu, Al, and ITO electrodes, respectively. The silver electrode-based device exhibits the highest output performance, with significantly enhanced *V*_OC_, *I*_SC_, and *Q*_SC_ compared to other materials, highlighting its superior conductivity and charge collection efficiency.

**Fig. S7** Influence of device size on signal acquisition performance. **(a)** Photographs of the fabricated e-skin patches with different sizes (3×3, 4×4, 5×5, and 6×6 cm²). **(b)** Corresponding *V*_OC_ signals recorded during articulation of the phrase “hao jiu bu jian”. Smaller patches (3×3 and 4×4 cm²) show low-amplitude, distorted signals that fail to resolve syllable-related peaks. The 5×5 cm² patch yields improved signal quality, while the 6×6 cm² patch fully captures clear, syllable-correlated waveforms, demonstrating that sufficient size coverage is critical for reliable lip movement signal acquisition.

**Fig. S8** Flexibility characterization of the E-skin patch. Digital photographs show the device under different bending states, demonstrating its excellent mechanical flexibility and deformability.

**Fig. S9** Output performance under different bending conditions. The *V*_OC_ of the E-skin patch was measured when attached to cylinders with different radii of curvature (*R*). Under the flat state (infinite radius), the *V*_OC_ is 21.36 V, which increases to 24.91 V at *R* = 4.0 cm, 27.46 V at *R* = 3.0 cm, and reaches 29.0 V at *R* = 2.0 cm, indicating enhanced electrical output under higher curvature deformation.

**Fig. S10** Dynamic response characteristics and pressure sensitivity of the E-skin patch. **(a)** Response and recovery time of the device. The sensor exhibits a fast response time of 90 ms and a recovery time of 65 ms, enabling rapid tracking of lip muscle movements and meeting the real-time signal acquisition requirements for normal speech. **(b)** Pressure sensitivity of the device across a wide pressure range. In the low-pressure region (0–20 kPa), the sensor shows a high sensitivity of 3.70 kPa⁻¹, allowing detection of subtle pressure changes from lip movements. In the high-pressure region (20–60 kPa), the sensitivity is 0.60 kPa⁻¹, demonstrating stable performance and good adaptability to varying pressures.

**Fig. S11** *V*_OC_ and corresponding audio signals of the phrases “hao jiu bu jian” and “long time no see” at three speech speeds (0.5×, normal, 1.5×). **(a)** The *V*_OC_ signals exhibit distinct peak patterns corresponding to each syllable at different speeds. At 0.5× speed, the signal peaks are more dispersed and lower in amplitude, reflecting slower lip movements and weaker triboelectric charge generation. At normal speed, four clear voltage peaks align with the four syllables of the phrase. At 1.5× speed, the peaks become more compact and higher in amplitude, indicating faster lip motion and enhanced charge transfer efficiency, while the temporal sequence of peaks remains consistent with the syllable order. **(b)** Similar trends are observed for the English phrase. At 0.5× speed, four distinct but lower-amplitude peaks correspond to each word. At normal speed, four moderate-amplitude peaks match the phrase’s syllable rhythm. At 1.5× speed, the peaks are more concentrated and higher in amplitude, with preserved temporal spacing that reflects an accelerated but recognizable speech rhythm.

**Fig. S12** Time-frequency analysis of triboelectric signals and corresponding audio spectrograms for the phrases “hao jiu bu jian” and “long time no see”. The time-frequency energy distribution of the triboelectric signals shows high consistency with the audio spectrograms. At syllable boundaries, such as the transition between “hao” and “jiu”, both types of signals displayed pronounced energy spikes and rapid shifts in frequency components. During sustained vowel phases, exemplified by the /aɪ/ sound in “jian,” both signals showed stable concentration of low-frequency energy.

**Fig. S13** Dynamic time warping (DTW) analysis of silent speech signals. The DTW distance between two repetitions of the same phrase is 1.42, which is significantly lower than the DTW distance (2.71) between different phrases. Signals from the same category exhibit high similarity, while signals from different categories are clearly distinguishable.

**Fig. S14** DTW distance heatmap of all 15 target words. Diagonal elements represent intra-class distances, while off-diagonal elements represent inter-class distances. The diagonal values are significantly smaller than the off-diagonal ones, demonstrating excellent repeatability, stability, and inter-class separability of the collected signals.

**Fig. S15** Signal reproducibility evaluation of the word “watermelon” over ten repeated trials. All recorded *V*_OC_ signals show highly consistent overall envelopes, with the first major peak corresponding to the syllable “wa-ter” appearing around 0.6–0.8 s, followed by stable secondary peaks for subsequent syllables. The positions, amplitudes, and morphologies of these peaks remained highly stable across trials. Quantitative analysis reveals an average peak timing error of only 11.2 ms, indicating excellent temporal synchronization accuracy across trials. Although the main peak amplitude varied slightly, the overall fluctuations were within a reasonable range, confirming reproducible energy distribution. The signal baseline was stable with no abnormal interference, further validating the reliability of data acquisition.

**Fig. S16** Mechanical stability and environmental adaptability of the E-skin patch. **(a)** Repeated attachment and detachment test over 30 cycles. The sensor retained 98% of its initial output after the first five cycles, which gradually declined to 96% after ten cycles, 93% after twenty cycles, and 88% after thirty cycles, demonstrating good reliability and repeatability under repeated use. **(b)** Simulated humidity tests under dry (~40% RH), mildly humid (~65% RH, simulated light sweating), and heavily humid (~85% RH, simulated heavy sweating) conditions. The output voltages were 3.88 ± 0.08 V (dry), 3.71 ± 0.09 V (mildly humid, 95.6% of dry value), and 3.42 ± 0.11 V (heavily humid, 88.1% of dry value).

**Fig. S17** Mechanical durability test of the E-skin patch over 10,000 press-release cycles. The contact-separation frequency was fixed at 1 Hz.

**Fig. S18** STFT time-frequency heatmaps of triboelectric signals and corresponding audio spectrograms for six representative target words (“blue”, “yellow”, “orange”, “purple”, “mutton”, and “sausage”).

**Fig. S19** STFT time-frequency heatmaps of triboelectric signals and corresponding audio spectrograms for six representative target words (“turkey”, “cucumber”, “bean”, “cabbage”, “grape”, and “watermelon”).

**Fig. S20** STFT time-frequency heatmaps of triboelectric signals and corresponding audio spectrograms for three representative target words (“breakfast”, “lunch”, and “dinner”).

**Fig. S21** Model training process diagram of the multi-layer neural network based on CNN.

**Fig. S22** Training and test accuracy curves of the CNN model across 12 repeated experiments with different random seeds.

**Fig. S23** Cross-subject and cross-time generalization performance of the silent speech recognition system. **(a)** Cross-subject evaluation results: Individual test accuracies for four participants were 98.90%, 98.63%, and 97.72%, with a cross-subject generalization accuracy of 97.15%. **(b)** Cross-time stability test results: recognition accuracies on days 1, 3, 7, and 10 were 98.73%, 98.44%, 97.84%, and 97.62%, respectively.

**Fig. S24** Performance evaluation of the system on continuous sentences. **(a)** Representative signal waveforms of the five tested sentences: "How are you", "Nice to meet you", "Can you help me please", "What is your favorite color", and "I would like to have a cup of coffee". Short sentences (e.g., "How are you") exhibit simple waveforms with distinct peaks, while long sentences (e.g., "I would like to have a cup of coffee") display complex multi-peak structures. **(b)** Detailed information table of the five tested sentences (word count and syllable count). **(c)** Confusion matrix of sentence recognition, showing that misclassifications among sentences are extremely rare. **(d)** Training and test accuracy curves, demonstrating rapid convergence of both accuracies with excellent stability and generalization capability. The average recognition accuracy across the five sentences is 97.3%.

**Fig. S25** Dialogue content and the real-time E-skin patch signals of the two characters in the lip language virtual communication system application.

**Fig. S26** DTW distance heatmap of 29 individual words extracted from the dialogue.

**Fig. S27** DTW distance heatmap of the four complete sentences in the dialogue.

**Table S1** Performance comparison of the proposed system with other silent speech recognition systems

| Sensor Type | Word Number | Machine learning methods | Accuracy | Power Supply | Channel Count | Refs. |
| --- | --- | --- | --- | --- | --- | --- |
| TENG-based | 15 | Multi-layer CNN | 98.75% | Self-powered | 1 | **This work** |
| TENG-based | 20 | dilated RNN | 94.50% | Self-powered | 1 | [S1] |
| TENG array | \ | ML | 97.06% | Self-powered | 4 | [S2] |
| TENG-based | 30 | CNN–LSTM | 95.83% | Self-powered | 1 | [S3] |
| sEMG | 33 | SVIT-SSR | 96.67% | Battery-powered | ≥8 | [S4] |
| sEMG | Large-scale | GMM+HMM | 91.1% | Battery-powered | 8 | [S5] |
| Wearable motion capture | 93 | TCN | 92.0% | Battery-powered | \ | [S6] |
| TENG-based | 12 | SVM | 96.30% | Self-powered | 1 | [S7] |

**Table S2** Summary of training and test accuracies across 12 repeated experiments

| **Experiments** | **Train Accuracy** | **Test Accuracy** |
| --- | --- | --- |
| 1 | 98.35773 | 98.6749 |
| 2 | 97.15447 | 98.8099 |
| 3 | 98.50407 | 98.6741 |
| 4 | 97.54472 | 98.7098 |
| 5 | 95.23577 | 98.7747 |
| 6 | 98.00000 | 98.7399 |
| 7 | 96.86179 | 98.6743 |
| 8 | 98.21138 | 98.8732 |
| 9 | 96.74796 | 98.8749 |
| 10 | 95.85366 | 98.6545 |
| 11 | 98.16260 | 99.5753 |
| 12 | 92.73171 | 99.6709 |
| Average | 96.94716 | 98.8922 |
| Standard deviation | 1.605701713 | 0.335344696 |

**Table S3** Performance comparison of different machine learning models on the silent speech recognition task. All models were evaluated under identical conditions using an 8:2 training/test split. Traditional machine learning models (SVM, Random Forest, KNN) achieved accuracies of 86.4%, 84.7%, and 79.2%, respectively, substantially lower than the proposed method (98.75%). LSTM and GRU achieved accuracies of 93.6% and 94.2%, respectively, still significantly lower than the proposed approach. The Transformer achieved 97.3% accuracy, approaching the proposed method's performance, but with O(*n*²) complexity. In contrast, the proposed four-layer CNN achieved 98.89% accuracy with superior training efficiency (2.4 s/epoch) and a lower parameter count (≈2.3 × 10⁶) than the Transformer.

| Model | Accuracy | Number of parameters | Inference time (ms) | Type |
| --- | --- | --- | --- | --- |
| SVM | 86.4% | ~2.3×10⁴ | 2.1 | Traditional machine learning |
| Random Forest | 84.7% | ~1.5×10³ | 1.8 |  |
| KNN | 79.2% | — | 5.6 |  |
| LSTM | 93.6% | ~1.1×10⁶ | 3.2 s/epoch | Recurrent neural network |
| GRU | 94.2% | ~0.9×10⁶ | 2.8 s/epoch |  |
| Transformer | 97.3% | ~3.1×10⁶ | 4.1 s/epoch | \ |
| **This work** | 98.75% | ~2.3×10⁶ | 2.4 s/epoch | \ |

**Supplementary Movies**

**Movie S1** Triboelectric Nanogenerator-based lip language display system.

**Movie S2** Triboelectric Nanogenerator-based lip language-controlled game.

**Movie S3** Triboelectric Nanogenerator-based virtual communication.

**Supplementary References**

[S1] Y. Lu, H. Tian, J. Cheng, F. Zhu, B. Liu et al., Decoding lip language using triboelectric sensors with deep learning. Nat. Commun. **13**(1), 1401 (2022). <https://doi.org/10.1038/s41467-022-29083-0>
[S2] P. Parashar, L.-C. Shen, Y.-H. Lee, M.K. Sharma, B.K. Nahak et al., A highly flexible self-powered triboelectric sensor array for silent speech recognition and swallowing motion analysis (small 36/2025). Small **21**(36), e70436 (2025). <https://doi.org/10.1002/smll.70436>
[S3] S. Lin, Y. Guo, X. Zeng, X. Zhou, Y. Zhang et al., TENG-based self-powered silent speech recognition interface: from assistive communication to immersive AR/VR interaction. Nanomicro Lett. **18**(1), 143 (2026). <https://doi.org/10.1007/s40820-025-01982-z>
[S4] Z. Li, B. Ma, W. Mao, J. Zhang, Z. Yu et al., SVIT-SSR: a sEMG-based vision transformer approach for silent speech recognition. Electron. Lett. **60**(21), e13285 (2024). <https://doi.org/10.1049/ell2.13285>
[S5] G.S. Meltzner, J.T. Heaton, Y. Deng, G. De Luca, S.H. Roy et al., Development of sEMG sensors and algorithms for silent speech recognition. J. Neural Eng. **15**(4), 046031 (2018). <https://doi.org/10.1088/1741-2552/aac965>
[S6] S. Liu, T. Fawden, R. Zhu, G.G. Malliaras, M. Bance, A data-efficient and easy-to-use lip language interface based on wearable motion capture and speech movement reconstruction. Sci. Adv. **10**(26), eado9576 (2024). <https://doi.org/10.1126/sciadv.ado9576>
[S7] H. Zhou, D. Li, X. He, X. Hui, H. Guo et al., Bionic ultra-sensitive self-powered electromechanical sensor for muscle-triggered communication application. Adv. Sci. **8**(15), 2101020 (2021). <https://doi.org/10.1002/advs.202101020>
